# EGG CONSUMPTION AND INCIDENT CARDIOVASCULAR DISEASE: VARIATION BY DIETARY PATTERN

**DOI:** 10.1093/geroni/igad104.3295

**Published:** 2023-12-21

**Authors:** Donna Kritz-Silverstein, Blake Blake Anuskiewicz

**Affiliations:** University of California San Diego, San Diego, California, United States; University of California San Diego, San Diego, California, United States

## Abstract

Some studies link egg consumption with cardiovascular disease, but none considers modification by dietary pattern. This study examines effects of egg consumption on incident cardiovascular disease (CVD), and variations of associations within the context of dietary pattern. Participants were 4,930 men and women from the Rancho Bernardo Study without prevalent CVD in 1972-74, who were followed through 2021 via mailed surveys, follow-up visits, and death certificates. Dietary data from 1988-91 enabled calculation of the alternate Mediterranean diet (aMed) score for 1632 individuals. Comparisons showed those with incident CVD (N=2066, 49.1%)were older (p< 0.001), had higher BMI (p=0.004), cholesterol, triglycerides, systolic and diastolic blood pressures (p’s< 0.001) than those without incident CVD, but did not differ in egg consumption. Those consuming more eggs/week were older (p< 0.001) and had higher BMI (p=0.002). Men consumed more eggs/week than women (means=4.2 vs. 3.2, p< 0.001). Adjusted Cox proportional hazards models showed there were no significant associations of egg consumption with incident CVD in men or women (HR=1.00, 95%CI=0.98-1.02; HR=1.01, 95%CI=0.99-1.03, respectively). However,analyses by dietary patterns showed that in men, but not women, increased egg consumption was associated with a 7% reduced risk of incident CVD (HR=0.93; 95%CI=0.88-0.98) among those with high aMed scores, but a 6% increased risk (HR=1.06; 95%CI=1.01-1.11) among those with low aMed scores. In conclusion, for men, dietary pattern modified associations such that among those with a healthier dietary pattern, egg intake was associated with lower incident CVD. For women, egg intake was unassociated with incident CVD regardless of dietary pattern.

